# NMR-Based Metabolomics in Metal-Based Drug Research

**DOI:** 10.3390/molecules24122240

**Published:** 2019-06-15

**Authors:** Federica De Castro, Michele Benedetti, Laura Del Coco, Francesco Paolo Fanizzi

**Affiliations:** 1 Dipartimento di Scienze e Tecnologie Biologiche ed Ambientali, Università del Salento, Via Monteroni, I-73100 Lecce, Italy; federica.decastro@unisalento.it (F.D.C.); laura.delcoco@unisalento.it (L.D.C.); 2 Consorzio Interuniversitario di Ricerca in Chimica dei Metalli nei Sistemi Biologici (CIRCMSB), via Celso Ulpiani, 27, 70126 Bari, Italy

**Keywords:** metabolomic, ^1^H NMR Spectroscopy, antitumour drugs, metal drugs, platinum drugs

## Abstract

Thanks to recent advances in analytical technologies and statistical capabilities, the application field of metabolomics has increased significantly. Currently, this approach is used to investigate biological substrates looking for metabolic profile alterations, diseases markers, and drug effects. In particular, NMR spectroscopy has shown great potential as a detection technique, mainly for the ability to detect multiple (10s to 100s) metabolites at once without separation. Only in recent years has the NMR-based metabolomic approach been extended to investigate the cell metabolic alterations induced by metal-based antitumor drug administration. As expected, these studies are mainly focused on platinum complexes, but some palladium and ruthenium compounds are also under investigation. The use of a metabolomics approach was very effective in assessing tumor response to drugs and providing insights into the mechanism of action and resistance. Therefore, metabolomics may open new perspectives into the development of metal-based drugs. In particular, it has been shown that NMR-based, in vitro metabolomics is a powerful tool for detecting variations of the cell metabolites induced by the metal drug exposure, thus offering also the possibility of identifying specific markers for in vivo monitoring of tumor responsiveness to anticancer treatments.

## 1. Introduction

In international scientific researches, the term “Omic” started to be used in the 1990s, when the Human Genome Project, whose aim was to determine the DNA nucleobases’ sequences, was launched, in order to identify and map genes of the human genome (the “genomics” research field) [1]. The growth of scientific knowledge and the development of new technologies increased the need to further delimit and define the fields of related research, mainly focusing on the detection of mRNA (transcriptomics), proteins (proteomics), and metabolites (metabolomics) in specific biological samples [2].

These research strategies resulted in many applications and still have much potential. In fact, Omics technologies can be applied, not only for the understanding of biological systems in a normal physiological state, but also to get insight for specific disease conditions. Indeed, these technologies can play a determinant role in screening, diagnosis, and prognosis, as well as in the understanding of diseases etiology [2].

“Metabolomics” is the latest Omics technology. Like the other Omics sciences (such as genomics, transcriptomics, and proteomics), metabolomics uses analytical technologies that allow the production of large amounts of information (big data). In the specific case of metabolomics, the analytical techniques are normally used to identify, characterize, and quantify small biological molecules that are involved in the structure, function, and dynamics of cells, tissues, or organisms [3]. In this respect, different from the others Omics sciences, which can only describe what might happen to a biological system in the future, metabolomics gives information about what happened in the considered system and producing the observed metabolites, thereby getting direct insight about the physiological status of the studied organism [4]. 

The importance of metabolic profiling of bio-fluids was recognized, for the first time, in the late 1990s with the introduction of the term “metabonomic”. This was last used to describe the quantitative measure of the metabolic, multi-parametric, and time-correlated response that a living system gives to a (patho) physiological stimuli or to genetic modifications [5]. Instead, the later definition of “metabolomics” consists of the identification and quantification of all the main low-molecular-weight metabolites/intermediates that vary according to the physiological or pathological state of the cell, tissue, organ, or organism of a biological system [6,7,8,9]. However, although some confusion has crept into the field, the difference in the use of metabolomics and metabonomics over the recent years has been clearly described [10], and nowadays the two terms are often used interchangeably [11].

Metabolomic science was born with the important aims of (among others) population profiling (for identifying metabolome-wide associations and novel risk biomarkers), food material profiling (for identify origin and for a quality control), and individual profiling (for a personalized health care). Nevertheless, it is also currently used for studying the mechanism of drug toxicity/efficiency and to develop new drugs [12]. On the other hand, there are some limitations related to the use of the technique, mainly due to biological variance and environmental influence, the difficulty of measuring minor (low-concentration) metabolites and the complexity of the resulting data sets. Despite these limitations, there are many advantages in using a metabolomic approach. First of all, there is the lack of need for analyte pre-selection, but also the achievable robustness and stability of analytical platforms [10].

It is important to consider that metabolomics generally consist of the use of separation (Gas Chromatography, GC, Capillary Electrophoresis, CE, High Performance Liquid Chromatography, HPLC, Ultra High Performance Liquid Cromatography, UPLC) and detection techniques (Nuclear Magnetic Resonance, NMR, and Mass Spectrometry, MS). Traditional methods require separation and optimization of the separation condition each time, followed by identification. Often, multiple slow separations (up to 72 h per sample) are necessary, and intensive manual work is needed, in addition to constant supervision and high-level skills [7,10]. In this context, the use of NMR spectroscopy in metabolomics studies has been given high throughput, thanks to the potential of this specific technique. Firstly, there is the advantage of measuring multiple (10 to 100) metabolites at once, with no need of their physical separation and a very limited sample working up. Secondly, this technique is non-destructive, and can be quantitative (different from MS) but capable at the same time, to allow metabolic profiles or “fingerprint” collection of the examined biological samples. 

Due to the recent strong improvements of NMR technique and technologies, the NMR-based metabolomic is becoming a fast-growing and powerful technology [7]. Such improvements are essentially related to more reliable spectrometers, especially highly-sensitive NMR probes, with versatile acquisition sequences that can allow faster performance and substantial NMR experiments. Both the development of powerful databases of metabolic data and efficient multivariate statistical methods have simplified the high complexity (number of spectra, number of groups, number of condition) of big data set handling. Nowadays, all these aspects have made NMR-based metabolic profiling an unbiased tool that can provide fully quantitative data for most of the components in a complex mixture [7]. 

## 2. NMR Metabolomics in Cancer

One of the biggest areas of metabolomic research has been the discovery of metabolic biomarkers and biopatterns testifying to alterations due to cancer [4]. Cancer is, in fact, a fatal malignancy worldwide, as reported in the results of the Global Cancer Statistics of the International Agency for Cancer Research (IARC). In 2018, 18.1 million new cancer cases were estimated worldwide, as well as about 10 million deaths, over a half of cancer patients. These estimated data fully justify the close attention of researchers for improved technologies, allowing not only studies on cancer progression, but also the discovery of new biomarkers for a rapid and early diagnosis [13].

**Figure 1:**
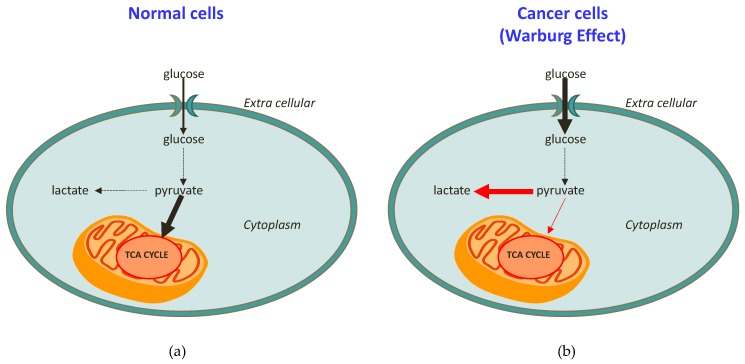
Schematic representation of (**a**) healthy cell metabolism in comparison to (**b**) cancer cell metabolism (the Warburg effect).

At this regard, it was demonstrated that a hallmark of cancer is represented by a reprogrammed metabolism. This was reported for the first time in the 1920s, thanks to the pivotal research of Otto Warburg showing that the metabolic patterns of cancer cells are generally different from those of normal cells [14]. In brief, normal cells use mitochondria to oxidise glucose and convert glucose into lactate only under hypoxia conditions (Figure 1a). In contrast, cancer cells avidly consume glucose, even in the presence of oxygen, and this is strictly associated with a lactate production increase (and then excretion; Figure 1b), together with the consequent acidic pH of cancer cells [14,15,16]. This discovery, at that time defined as “the root cause of cancer”, and subsequently simply indicated as “the Warburg effect”, won him the Nobel Prize for Medicine [15,16]. By increasing knowledge and scientific research, it was therefore proved that the Warburg effect cannot really be considered “the root cause of cancer”, today known to be determined by a combination of genetic and environmental triggering factors [17]. Nevertheless, the Warburg effect clearly describes how cellular respiration enzymes act in tumour cells, thereby opening new perspectives in the study of cancer [18]. In addition to the better understanding of cancer cells’ physiology, some studies are specifically designed to “reverse the Warburg effect” [19]. This last approach is also believed to be a valid method for pharmacologically acting against cancer. Indeed, several works have targeted the increased glycolysis, with the aim of inhibiting lactate production and excretion [19,20,21,22,23], and have found some success in preclinical models [24,25,26]. 

The Warburg effect has been confirmed in a variety of tumor types, including colorectal, breast, ovarian, lung, and glioblastoma cancers [14,18,27,28,29,30,31,32]. Furthermore, many studies have demonstrated that cancer cells are also dependent on fatty acid synthesis and glutaminolysis for proliferation [33]. All these differences with respect to normal cells suggest that targeting metabolic dependence could be a selective approach to treat cancer patients [27].

In this context, NMR spectroscopy, including in vivo magnetic resonance spectroscopy (MRS) and high-resolution magic angle spinning (HR-MAS NMR) analysis of tissue extracts, has been widely used to distinguish between different cell lines and tumor types [34]. As nuclear magnetic resonance spectroscopy is a high-throughput technology, it can be used to profile systemic metabolism in tumor diagnosis and prognosis [34]. NMR-based metabolomics have also been used to identify biomarkers for cancer, including hepatocellular carcinoma (HCC), colon cancer, ovarian cancer, breast cancer, prostate cancer, colorectal cancer, and others [4,35,36,37,38,39,40,41]. The aims of the studies have been to shorten the time to diagnosis, especially in cancers for which early detection and screening are difficult but would significantly affect therapeutic treatment decisions, as well as prognosis.

NMR metabolomics studies of cancer are applied to different kind of samples, such as bio-fluids (e.g., urine, whole blood, serum, plasma) and tissue/cell extracts (e.g., liver, brain, or kidney). Samples are normally examined directly, without extensive sample preparation, so that beside in vivo and in situ, a huge number of bio-fluids information are easily obtained [4,42,43,44,45,46,47,48,49,50,51].

**Figure 2:**
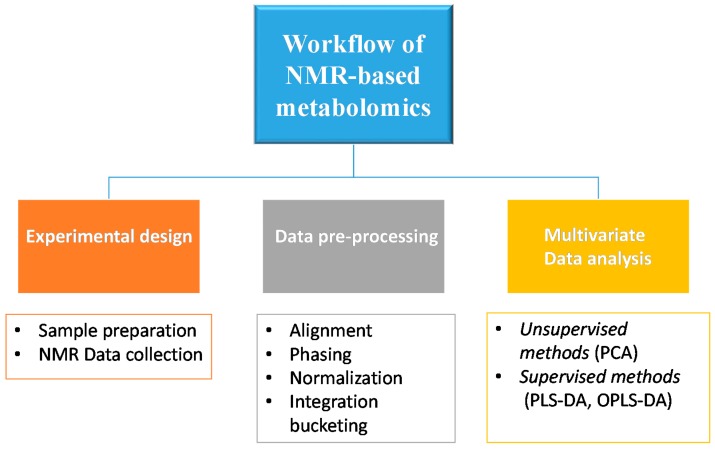
Schematic representation of the basic workflow of NMR-based metabolomics.

The basic workflow of an NMR-based metabolomic approach starts with experimental design, including the evaluation of appropriate sample numbers to yield informative and statistically significant results, as well as biological and analytical experiments, such as sample preparation and NMR data acquisition. After this, the data preprocessing phase and subsequent data analysis follow. Then the metabolomics study requires the interpretation of the results using a multivariate data analysis, which usually consists of unsupervised and supervised methods (Figure 2).

## 3. Multivariate Data Analysis

High-resolution NMR spectroscopy allows the detection of a wide range of chemical species simultaneously in a single experiment, by obtaining a large amount of data. For this reason, these spectroscopic techniques are generally coupled to a multivariate statistical treatment. Among available statistical methods, the most used in the NMR-based metabolomic studies were unsupervised principal component analysis (PCA), supervised partial-least-squares discriminant analysis (PLS-DA), and orthogonal partial-least-squares discriminant analysis (OPLS-DA).

Principal components analysis (PCA) has been widely used in several metabolomic studies, as it simply describes the intrinsic variation in the spectral data using a smaller number of factors or principal components (PCs). Each PC is a linear combination of the original data (the NMR variables), explaining the maximum amount of variance, not accounted by the previous PCs. Conversion of the spectral data to PCs results in two matrices, known as scores (corresponding to samples) and loadings (corresponding to NMR variables) [52]. By this unsupervised approach, it is possible to describe the general trend of samples and their potential clustering, depending on specific loadings (spectral variables) that may cause any cluster separation. In addition, there are other many unsupervised methods (e.g., *k*-means and hierarchical cluster analysis) also used to study how the data is distributed and to define groups within the samples. These methods essentially work on data forming a tree diagram, or dendrogram, which shows the relationships between samples. In general, the unsupervised analytical methods were used with the aim of identifying patterns in the data, as well as to obtain a general overview of the multivariate profiles. The further-supervised multivariate data analytical tools, such as projection to latent structure (PLS) and orthogonal projection to latent structure (OPLS), are tightly focused on the effects of interest, using any sample of a priori knowledge (e.g., class membership). Specifically, these methods relate the data matrix containing independent variables, such as spectral intensity values (defined as the X matrix), to a matrix containing dependent variables (e.g., measurements of response, such as toxicity or drug treatment) for those samples (Y matrix) [52,53]. This last (Y matrix) can be defined by continuous (pH values or the time course of the progression of a pathological effect), or discrete variables (group identity, health condition). In this case, a PLS discriminant analysis (PLS-DA), or its recent modification (OPLS-DA), is performed to examine the intrinsic variation in the data, as well as for classification purposes. In particular, the use of OPLS-DA consists in the separation of the X data matrix (containing independent variables, such as spectral intensities) into two parts, one that is linearly related to Y and one that is unrelated (orthogonal) to Y. This partitioning of the X data facilitates model interpretation and its potential use in the prediction of new samples. The statistical model performance (robustness and predictive capability) is evaluated through permutation tests and cross-validation, together with the R^2^ and Q^2^ indices. The last two parameters (R^2^ and Q^2^) respectively describe the total variations in the data and the predictability of models [53,54,55,56].

## 4. The Potential of an NMR Metabolomics Approach in Monitoring the Response to Metal-Based Antitumor Drugs

The development of a new drug is a fundamental step in medicine. It consists in identifying a general therapeutic area of interest, a specific disease to treat, and in most cases, the biological “target” [57]. In the past, the lack of complete knowledge of the action mechanism for a specific drug often resulted in the failure of clinical trials, as in the well-known case of Dimebon, a drug studied for Alzheimer’s [58]. In fact, the Food and Drug Administration (FDA), the U.S. government agency responsible for regulating food and pharmaceutical use, does not require any specific understanding about the drugs’ mechanism of action before starting clinical trials. Nevertheless, the last aspect is an essential step in the development of a new drug, because a clear understanding of the altered metabolic pathways due to a pharmacological treatment, as well as the action mechanism of a drug before it enters clinical trials, may prevent a late-stage failure [57]. A good strategy to detect treatment-related altered metabolic pathways is the monitoring of changes in the metabolome following the starting of chemotherapeutic treatment [59]. Indeed, the response to chemotherapy is often known to induce several metabolic alterations with respect to the physiological condition [59].

One of the most widely used anticancer drugs is cisplatin, a metal-based drug that acts by binding to genomic DNA targets. The discovery of cisplatin, a Pt(II) compound, by Barnett Rosenberg in 1960 represents a milestone in the history of metal-based compounds in the treatment of cancer [60]. Currently, cisplatin is used for the clinical management of patients affected by testicular, ovarian, head and neck, colorectal, bladder, and lung cancers. 

Recently, many more metal-based compounds have been synthesized, either by redesigning the cisplatin chemical structure through ligand substitution, or by building completely new metal-based compounds. Therefore, regarding to pre-established structure−activity relationships (SARs), many unconventional Pt(II) and Pt(IV) compounds have been tested [61]. Nevertheless, other anticancer metal complexes—for example, complexes containing ruthenium (Ru(II) and Ru(III)) [62,63]), titanium (Ti(IV)), and gold (Au(I) and Au(III))—have been studied [64]. In particular, Palladium (Pd) compounds have drawn particular attention, due to its similarity to Pt(II) (electronic structure and coordination chemistry) in showing favourable cytotoxic activity, despite both of their high lability [65,66,67]. Involved studies have essentially developed new metal-based drugs with the aim of enhanced safety and cytotoxic profiles, but also to overcome the resistance phenomena observed for cisplatin [68]. 

The use of NMR-based metabolomic approaches in the study of metal-based anticancer drugs is very new. Indeed, few papers, and only in recent years, have dealt with the use of this tool by studying the response of biological systems (biofluids, cells, and tissue) to metal-based drugs treatment in vitro and in vivo. This work covers the state-of-the-art survey of the NMR-based metabolomics approach adopted in metal-based drugs research, focusing on the possible applications and advantages of this investigation approach.

### 4.1. NMR Metabolomics Studies of FDA-Approved Metal Complexes

**Figure 3:**
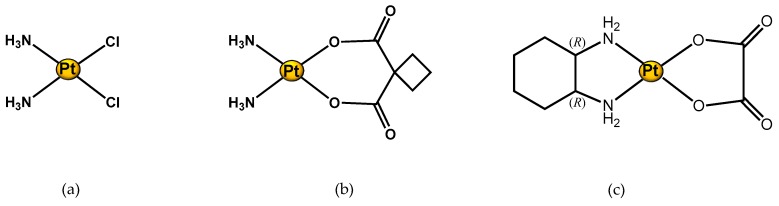
Structure of Pt(II) complexes studied through an ^1^H NMR metabolomic approach. (**a**) Cisplatin; (**b**) Carboplatin; and (**c**) Oxaliplatin.

As expected, cisplatin is the first metal-drug studied by using an NMR metabolomic approach (see Figure 3a). Different samples, such as biofluids (urine, serum), and different cancer cell lines were examined in order to investigate cisplatin-induced metabolic alteration under different conditions of treatment, and in different tumour types. In particular, one of the interest topics of these scientific studies has been cisplatin-induced side effects. 

At this regard, Portilla et al. have applied NMR-based metabolomic technology to the study of cisplatin-induced nephrotoxicity (one of the cisplatin-induced side effects). The research was performed through the analysis of urine samples of mice treated with cisplatin. Following administration of cisplatin for three days, the samples were collected and analysed by means of ^1^H NMR spectroscopy coupled to multivariate data analysis (MVA). This study demonstrated that the presence of glucose, amino acids, and Kreb’s cycle metabolites in the urine after cisplatin administration indicates the development of renal failure. The last specific cisplatin-induced metabolic NMR profile of urine in mice developing acute renal failure suggests that an injury-induced metabolic profile could be a biomarker of cisplatin-induced nephrotoxicity [69]. 

In further studies, Wen et al. extended the work of Portilla et al. to a larger number of animals, using a statistical approach by taking into consideration the intra-group variation (experiments are performed on the urine of 22 male Sprague–Dawley rats treated with cisplatin). Using NMR-based metabolomics combined with orthogonal partial least squares-discriminant analysis (OPLS-DA), the authors identified glucose, glycine, taurine, and branched amino acids as urinary biomarkers strictly associated with the renal toxicity induced by cisplatin [70].

As other known platinum-based drugs (such as carboplatin (Figure 3b) and oxaliplatin (Figure 3c)) approved by the FDA are largely used in the treatment of different tumours. In particular, oxaliplatin is used for the treatment of advanced colorectal cancer, though it can cause painful peripheral neuropathies, which pathophysiology has not been fully clarified. For this reason, Ferrier et al. used NMR metabolomics to monitor and investigate the effects of a polyamine-deficient diet in an animal model of oxaliplatin-induced acute pain hypersensitivity. The authors compared samples extracted from rat spinal dorsal horn, from rats fed with a normal diet, oxaliplatin-treated rats fed with a normal diet, and oxaliplatin-treated rats fed with a polyamine-deficient diet. Interestingly, they observed an increase of glutamate concentration in the spinal dorsal horn of rats after oxaliplatin treatment, as well as the ability of a polyamine-deficient diet to prevent neurotoxicity. This type of study is of crucial importance in oncology, especially taking in consideration that this type of injury (neurotoxicity) is often the cause of dose reduction or of discontinued treatment in cancer patients [71].

NMR-based metabolomics has also proved to be a useful tool in the prediction of treatment response to chemotherapy. The last is the principal aim of Jiang et al.’s research [72]. In this case, the experiments were focused on finding metabolites whose basal levels can predict the treatment response of 29 metastatic breast cancer (MBC) patients to gemcitabine–carboplatin chemotherapy. To make this, the ^1^H NMR spectra of serum samples from patients with varying responses to subsequent chemotherapy were proved to be essential. Significantly, lower baseline levels of serum formate and acetate were observed in breast cancer patients who progress (MBC), with respect to those who achieve clinical benefits after receiving gemcitabine–carboplatin (GC) chemotherapy. These results indicated that formate and acetate might be used to select patients who should not be treated with GC chemotherapy, to avoid unnecessary adverse side effects.

Another NMR metabolomic application concerned with the possibility of distinguishing patients who are sensitive or insensitive to chemotherapy. In the interesting research of Xu et al., the serum of patients undergoing a platinum-based combined chemotherapy for lung cancer was analysed. The work demonstrated that insensitive subjects can be identified from the sensitive ones by observed up-regulation of glucose and taurine, but reduced alanine and lactate concentrations in the serum. The serum metabolic profile of patients can be therefore a valuable tool of their response to platinum-based combination chemotherapy [73].

In addition to the analysis of bio-fluids with NMR-metabolomics, the analysis of tumour cells gave high throughput regarding a drug action mechanism or for peculiar metabolites expressed in a specific type of tumour. This could be defined as the first step toward understanding drug synergism and a definition of new drug efficacy biomarkers. Duarte et al. deeply investigated the cisplatin treatment response in human lung cells and MG-63 osteosarcoma cells (in the last cell line, considering the comparison with doxorubicin and methotrexate as well) [74,75]. Their research strongly supports the ability of NMR metabolomics to measure cellular responses to different drug treatments, confirmed by the found potential biomarkers of cisplatin treatment response in the considered tumour cell lines.

Moreover, the dose-responsive effects of a metal-based drug could be also defined by NMR metabolomics. An example is the investigation of cisplatin response in human liver (L02) cells, described by Liu et al. [76]. Through the OPLS-DA analysis of data obtained from ^1^H NMR spectra of L02 cell extracts (from control and cisplatin-treated groups), a clear discrimination was found. The study strongly supports the advantage of a global analysis using metabolomics, in order get an insightful view into the dose-response relationship, rather than using a single endpoint at the molecular level.

### 4.2. NMR Metabolomic Studies of New Metal Complexes

**Figure 4:**
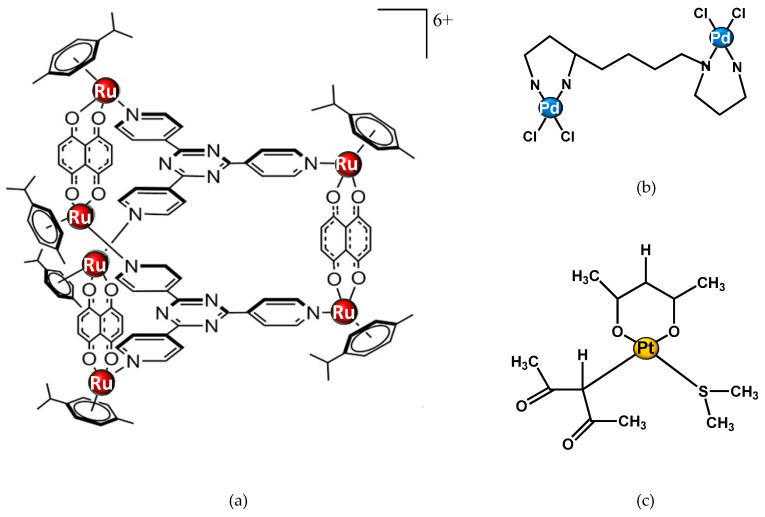
Structure of metal complexes different from cisplatin and studied through ^1^H NMR metabolomic approaches. (**a**) Ruthenium metallaprism; (**b**) Pd_2_spermine; (**c**) [Pt(*O*,*O*’-acac)(γ-acac)(DMS)], Ptac2S.

Through the NMR-metabolomics investigation of the effects of approved and widely used metal drugs (such as cisplatin, carboplatin, and oxaliplatin), this new field helps to discover how a new metal drug acts in a biological system, and how to define the specific target of the drug. An example is the study of Vermathen et al. on a new ruthenium complex. They used this technique to profile cells in response to treatment with a hexacationic ruthenium metallaprism (Figure 4a) in three different cell lines: A2780 (human ovarian cancer cells), A2780cisR (cisplatin resistant cells), and HEK-293 (human embryonic kidney cells). This research revealed a different response depending both on the cell type and incubation time. The observed different metabolic profile is mainly due to changes in level expression of lipids, choline-containing compounds, glutamate and glutathione, nucleotide sugars, lactate, and some amino acids. The time-dependent metabolic response patterns suggest that A2780 cells on the one hand, and HEK-293 and A2780cisR cells on the other, may follow different cell-death pathways and exist in different temporal stages thereof [77].

Other NMR-metabolomics studies have focused on Palladium (Pd) compounds. Iamego et al. describes metabolomic studies of a Pd_2_Spermine (Pd_2_Spm) complex (Figure 4b) on osteosarcoma MG-63 and osteoblastic HOb cells. The aim of their work was to assess the impact of the potential palladium drug on cell metabolism in comparison with cisplatin. The authors have shown that despite its higher cytotoxicity, Pd_2_Spm induced a lower (and reversible) metabolic impact on MG-63 cells, as well as the absence of apoptosis; however, it induced significant deviations in the osteoblastic amino acid metabolism. Moreover, when administrated in combination with doxorubicin and methotrexate, the complex Pd_2_Spm induced strong metabolic deviations in lipids, choline compounds, amino acids, nucleotides, and compounds related to anti-oxidative mechanisms (e.g., glutathione, inositol, and hypoxanthine). These results were similar to those observed for cisplatin treatments in combination with doxorubicin and methotrexate. These findings open promising perspectives related to the impact of Pd_2_Spm on osteosarcoma cellular metabolism, particularly in drug combination protocols. The lipid metabolism, glycosylation, and amino acid metabolisms have emerged as relevant features for targeted studies, to understand further the potential anticancer mechanism of combined Pd_2_Spm [67].

NMR metabolomics can be applied also in the study of drug treatment resistance. Recently, our group reported an ^1^H-NMR-based metabolomic study to evaluate the response of a cisplatin-resistant epithelial ovarian carcinoma cell line, SKOV-3, to a new promising Pt(II) drug, [Pt(*O,O*′-acac)(γ-acac)(DMS)], or Ptac2S (Figure 4c). This complex is interesting for its higher anticancer activity, enhanced pharmacokinetics, bio-distribution, and tolerability showed in comparison to cisplatin. This is produced by a non-genomic mechanism of action, as previously demonstrated both in vitro and in vivo [77,78,79,80,81,82]. The observed higher cytotoxicity of Ptac2S with respect to cisplatin in the cisplatin-resistant SKOV-3 cells, evaluated by a preliminary MTT (3-(4,5-dimethylthiazol-2-yl)-2,5-diphenyltetrazolium bromide) assay, led us to investigate further the action mechanism of this complex, which is able to overcome cisplatin resistance. 

**Figure 5:**
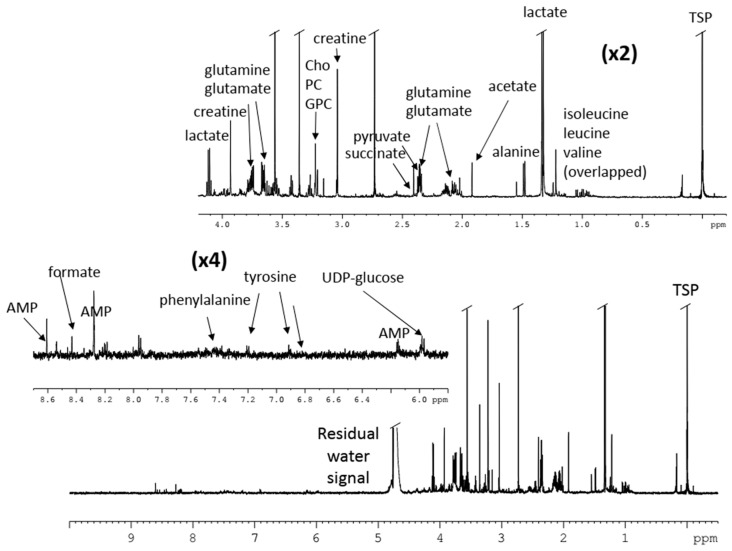
^1^H NMR spectrum of aqueous extracts of cisplatin-resistant, epithelial ovarian carcinoma cell line SKOV-3 cells treated with [Pt(*O*,*O*’-acac)(γ-acac)(DMS)], Ptac2S [81].

Cell extracts (aqueous, as shown in Figure 5, and lipidic), as well as the recovered culture media for each condition (controls, Ptac2S, and cisplatin-treated) and time of treatment (6–24 h), were analysed by ^1^H NMR. As expected, multivariate analysis of the NMR data gave new insights about the mechanism of action of Ptac2S. Through a careful investigation of the metabolites responsible for the observed differences between the two treatments (Ptac2S and cisplatin), it was found that the exposure of SKOV-3 cells to Ptac2S caused a cell membrane modification, a decrease of Krebs cycle efficiency, and an inhibition of the protein catabolism. By metabolomic analysis, the role of pyruvate was also found to be crucial in the antitumor activity of Ptac2S. The SKOV-3 cells’ metabolic behaviour after the Ptac2S treatment suggests a possible “reversal of the Warburg effect” through the inhibition of lactate synthesis (probably due to a modulation of the lactate dehydrogenase activity). Furthermore, the different lipidic profile in Ptac_2_S, with respect to cisplatin-treated cells, indicates a possible cell death mechanism different from apoptosis (the known cisplatin-induced mechanism of death) [81]. In this context, biological assays have recently demonstrated that Ptac2S induces autophagy in another cisplatin-resistant cancer cell line (Caki-1, a renal cancer cell line) [82]. These findings parallel the results derived through the NMR metabolomic analysis, confirming the power of the technique in the investigation of mechanisms of action.

## 5. Conclusions

In this review, the great potential and application of NMR-based metabolomics in the metal-based drug research field are discussed [67,69,70,71,72,73,74,75,76,77,81]. Despite pitfalls due to the variability of cell cultures and the environmental influence, NMR metabolomics have excellent reproducibility and quantitative accuracy. This is made possible for standardized experimental methods (generally referred to controls), as well as for the stability of analytical platforms, both with the powerful statistical databases of metabolic data and efficient multivariate statistical methods that have also simplified the high complexity of the dataset. The use of NMR-based metabolomic approaches in the investigation of a metal drug action mechanism or for assessing tumour response to anticancer metal agents is a recent, fast-growing tool. The future perspectives are even more interesting. Due to the low work-up required, high data reproducibility, and high throughput, NMR spectroscopy is an optimal detection technique in metabolomics studies. Indeed, metal drug-induced side effects, treatment response prediction (thus also eventually resistance), and action mechanism key information for known and new compounds could be easily obtained using NMR-based metabolomics. A greater use of this technique could lead to personalized medicine, thus allowing high-throughput screening of pharmacologically active molecules, as well as lower doses chemotherapy. Furthermore, NMR-based metabolomics could also play an important role in clinical trials, preventing or reducing unwanted side effects of metal anticancer drugs by the early detection of metabolic dysfunctions in bio-fluids.
